# Role of copper ionophore–induced death in immune microenvironment and clinical prognosis of ccRCC: An integrated analysis

**DOI:** 10.3389/fgene.2022.994999

**Published:** 2022-10-03

**Authors:** Shunyao Xia, Haixing Jia, Zhipeng Qian, Youcheng Xiu

**Affiliations:** ^1^ Department of Urology, First Affiliated Hospital of Harbin Medical University, Harbin, China; ^2^ School of Bioinformatics Science and Technology, Harbin Medical University, Harbin, China

**Keywords:** ccRCC, machine-learning, bioinformatics, immune microenvironment, precise medicine, CID

## Abstract

**Background:** Clear cell renal cell carcinoma (ccRCC) is a malignancy with a high incidence rate and poor prognosis worldwide. Copper ionophore–induced death (CID) plays an important role in cancer progression.

**Methods:** One training and three validation datasets were acquired from TCGA, GEO and ArrayExpress. K-means clustering was conducted to identify the CID subtypes. The ESTIMATE and CIBERSORT algorithms were employed to illustrate the immune microenvironment of ccRCC. LASSO Cox regression was applied to construct the CID feature-based prognostic model. The immunotherapy cohort was acquired from the literature to explore the potential risk scores for predicting immunotherapy responsiveness.

**Results:** Two CID-related cancer subtypes of ccRCC were identified that performed different immune microenvironment characteristics and prognosis. Based on the identified subtypes, we analyzed the biological heterogeneity and constructed a gene prognostic model. The prognostic model performed well in one training dataset, three validation datasets, and different clinical pathological groups. The prognostic model has a good potential for predicting cancer immune features and immunotherapy responsiveness.

**Conclusion:** CID plays an important role in the tumor microenvironment progression of ccRCC. The robust gene prognostic model developed can help predict cancer prognosis, immune features, and immunotherapy responsiveness.

## Introduction

Renal cell carcinoma (RCC) is the major prevalent urinary system malignancy, with more than 430000 cases diagnosed worldwide in 2020 (Sung et al., 2021). Among RCC subtypes, clear cell renal cell carcinoma (ccRCC) is the most common and lethal form (Jonasch et al., 2014). The current progression of comprehensive therapy strategies for ccRCC, such as tyrosine kinases inhibitors, mTOR inhibitors and immune checkpoint blockades, has significantly improved the prognosis of patients (Motzer et al., 2014, 2015; Choueiri et al., 2017). However, a non-negligible rate of patients remains non-responsive to cancer therapy and with severe side effects (Kennedy and Salama, 2020; Braun et al., 2021). Furthermore, approximately 30% of patients with ccRCC are diagnosed with metastatic cancer, for which effective therapy strategies are limited (Patard et al., 2011). Consequently, ccRCC is a major global public health concern. Therefore, given the high incidence rate and poor prognosis of ccRCC, developing a robust prognostic model to assist patient prognosis evaluation and reveal the underlying heterogeneity mechanism of ccRCC is urgently in demand.

Redox-active copper plays an essential role in maintaining cell homeostasis and takes part in various biological processes, including energy metabolism, biosynthesis, and antioxidant defense (Tsang et al., 2021). Though copper is indispensable for the normal physiological activity of cells, it can be cytotoxic. In 2022, Tsvetkov et al. (2022) revealed an unexpected cell death pattern triggered by copper in a tricarboxylic acid (TCA) cycle metabolism-related mechanism-copper ionophore–induced death (CID). Meanwhile, copper-related drugs, such as copper chelation, have great potential to be developed as a clinic anti-cancer therapy (Yin et al., 2016; Lopez et al., 2019). ccRCC is a cancer type characterized by significant TCA metabolic heterogeneity (Wettersten et al., 2017). As CID may contribute to the heterogeneous ccRCC formation, CID status may be developed as an indicator of the prognosis of patients with ccRCC.

This study conducted an integrated analysis to illustrate the multi-omics features of CID-related genes in ccRCC and identified two CID subtypes of ccRCC. Then, we analyzed the microenvironment heterogeneity across the two CID subtypes. Based on the two identified CID subtypes, we developed a prognostic model to predict patient prognosis, immune characteristics, and immunotherapy responsiveness using a machine-learning method. Our research presented an overview of the regulatory function of CID during ccRCC progression and developed a robust CID-based model to help evaluate the prognosis and immunotherapy suitability during clinical practice.

## Methods and materials

### Data collection

The genomic data and corresponding clinical information on kidney clear cell carcinoma (KIRC) of The Cancer Genome Atlas (TCGA) were downloaded from the University of California Santa Cruz (UCSC) Xena online tool (https://xenabrowser.net/). LOWESS normalized gene expression profile and quantile normalized, and the log2 transformed gene expression profile of KIRC (GSE29609 and GSE22541) with corresponding clinical information were downloaded from Gene Expression Omnibus (GEO, http://www.ncbi.nlm.nih.gov/geo). The log2 quantile normalized expression data of 101 KIRC samples were downloaded from ArrayExpress (https://www.ebi.ac.uk/arrayexpress/experiments/E-MTAB-1980/), and clinical information was obtained from *Sato et al.* (Sato et al., 2013). The expression and clinical data of IMvigor210 trial were accessed with R package “IMvigor210CoreBiologies”. IMvigor210 was a single-arm phase Ⅱ study investigating the anti-PD-L1 antibody agent atezolizumab in patients with metastatic urothelial cancer (mUCC) (Mariathasan et al., 2018). The KIRC samples of TCGA was used as training dataset due to large sample size for statistical accuracy, and complete gene expression and clinical information (*eg.* stage, grade, survival time). For validation, we chosen the gene expression datasets of KIRC samples with survival time and survival status or disease-free survival time. The samples in IMvigor210 dataset has responsive information for immunotherapy.

### Variation and expression correlation

Genes involved in copper ionophore–induced death (CID) were obtained from a study by Tsvetkov et al. (2022), including *FDX1*, *LIAS*, *LIPT1*, *DLD*, *DLAT*, *PDHA1*, *PDHB*, *MTF1*, *GLS*, and *CDKN2A*. Somatic mutation, copy number variation (CNV) alterations, and differential expression between tumor samples and normal samples of CID genes were demonstrated. The prognostic value of CID genes was analyzed with a univariable Cox proportional hazards regression model (Supplementary Table S1). Co-expression status of CID genes was analyzed by Pearson correlation analysis (Supplementary Table S2), and the correlation network was visualized using Cytoscape software.

### Identification of CID subtypes

K-means clustering is an unsupervised learning algorithm that groups data based on each point euclidean distance to a central point called centroid. K-means clustering was performed to identify two CID subtypes based on CID gene expression by R package “pheatmap”. Finally, a total of 197 samples were grouped into “Subtype A” and 329 samples were grouped into “Subtype B” Principle component analysis (PCA) was applied to explore the difference between Subtypes A and B based on CID gene expression. Kaplan-Meier survival analysis and log-rank test were used to analyze the difference in overall survival (OS) among the two subtypes.

### Analysis of tumor immune infiltration microenvironment

The ESTIMATE algorithm was applied to evaluate the immune and stromal scores of each KIRC sample in TCGA using R package “estimate.” The proportion of infiltration of 22 immune cells for TCGA KIRC samples was inferred with CIBERSORT algorithm using the web-based analytical tool (https://cibersort.stanford.edu/) (Newman et al., 2015). CIBERSORT estimates the abundances of specific cell types in a mixed cell population using a gene expression-based approach. We focused on mRNA expression of five immune checkpoints, including PD-1, PD-L1, CTLA4, CD47 and BTLA. The immune cytolytic activity (CYT) was calculated as the mean of GZMA and PRF1 expression according to *Rooney et al.* (Rooney et al., 2015). A one-sided Wilcoxon rank-sum test was used to analyze the differences between subtypes.

### Differentially expressed genes between subtypes and functional analysis

A total of 1448 DEGs with |log2FC| > 1 and FDR <0.001 were identified using R package “edgeR” between subtypes (Robinson et al., 2010). Pathway and process enrichment analysis for 344 DEGs with |log2FC| > 2 and FDR <0.001 was performed using the Metascape web-based tool (https://metascape.org/gp/index.html), including many ontology sources such as KEGG Pathway, GO Biological Processes, and Reactome Gene Sets and Canonical Pathways (Supplementary Table S3) (Zhou et al., 2019).

### Construction of the prognostic model

A univariable Cox proportional hazards regression model was performed to identify prognostic DEGs. A total of 80 DEGs with *p* < 0.001 were selected. The least absolute shrinkage and selection operator (LASSO) method was used for significant prognostic DEGs selection in a Cox regression model by fitting a generalised linear model via penalised maximum likelihood. We analysed the lambda value (λ) using the 10 fold cross-validation, between λmin that gives minimum mean cross-validated error or λ1se, that gives a model such that standard error (SE) is within one standard error of the minimum. The process was conducted using R package “glmnet” (Engebretsen and Bohlin, 2019). Finally, a risk score formula was calculated by considering the expression of 17 optimized genes and correlation estimated multivariate Cox regression coefficients using R package “survival” (Supplementary Table S4). The risk score was calculated as follows:

Risk score=Σ(Expi*coefi) (1)

### Survival analysis

Patients were classified according to the median of risk score. The log-rank test was used to assess the survival time difference between high-risk and risk score patients using R package “survival.” Additionally, a stratified analysis was performed to determine whether the risk score retained its predictive ability in different subgroups according to gender, age, T stage, N stage, M stage, tumor stage, and tumor grade. Kaplan-Meier plots were used to present the results. Chi-square tests explored the relationships between the risk score and clinical characteristics (Table 1).

**TABLE 1:** Baseline characteristics of patients in TCGA KIRC cohort

Characteristics	Whole cohort	High PSR_score	Low PSR_score	p
TCGA cohort	(*n* = 526)	(*n* = 263)	(*n* = 263)	
Gender				0.044
Male	342(65.02%)	182(69.2%)	160(60.84%)	
Female	184(34.98%)	81(30.8%)	103(39.16%)	
Age				0.087
<65 years	329(62.55%)	155(58.94%)	174(66.16%)	
>=65 years	197(37.45%)	108(41.06%)	89(33.84%)	
T-stage				9.1 e-11
T1	269(51.14%)	95(36.12%)	174(66.16%)	
T2	68(12.93%)	36(13.69%)	32(12.17%)	
T3	178(33.84%)	121(46.01%)	57(21.67%)	
T4	11(2.09%)	11(4.18%)	0(0%)	
N-stage				0.0023
N0	239(45.44%)	115(43.73%)	124(47.15%)	
N1	16(3.04%)	14(5.32%)	2(0.76%)	
M-Stage				2.0 e-10
M0	436(82.89%)	191(72.62%)	245(93.16%)	
M1	80(15.21%)	66(25.1%)	14(5.32%)	
Stage				3.1 e-06
I	263(50%)	91(34.6%)	172(65.4%)	
II	56(10.65%)	28(10.65%)	28(10.65%)	
III	122(23.19%)	75(28.52%)	47(17.87%)	
IV	82(15.59%)	68(25.86%)	14(5.32%)	
Grade				1.2 e-05
G1	14(2.66%)	1(0.38%)	13(4.94%)	
G2	224(42.59%)	81(30.8%)	143(54.37%)	
G3	205(38.97%)	112(42.59%)	93(35.36%)	
G4	75(14.26%)	67(25.48%)	8(3.04%)	

### Statistical analysis

A one-sided Wilcoxon rank-sum test was used to test the discrepancy between CID subtypes or high and low-risk groups. Patients were divided into high risk and low risk groups according to the median of risk score. All statistical analyses were performed using R version 4.1.2. *p* < 0.05 was considered statistically significant.

## Results

### Multi-omics level alterations of CID genes in KIRC

**FIGURE 1:**
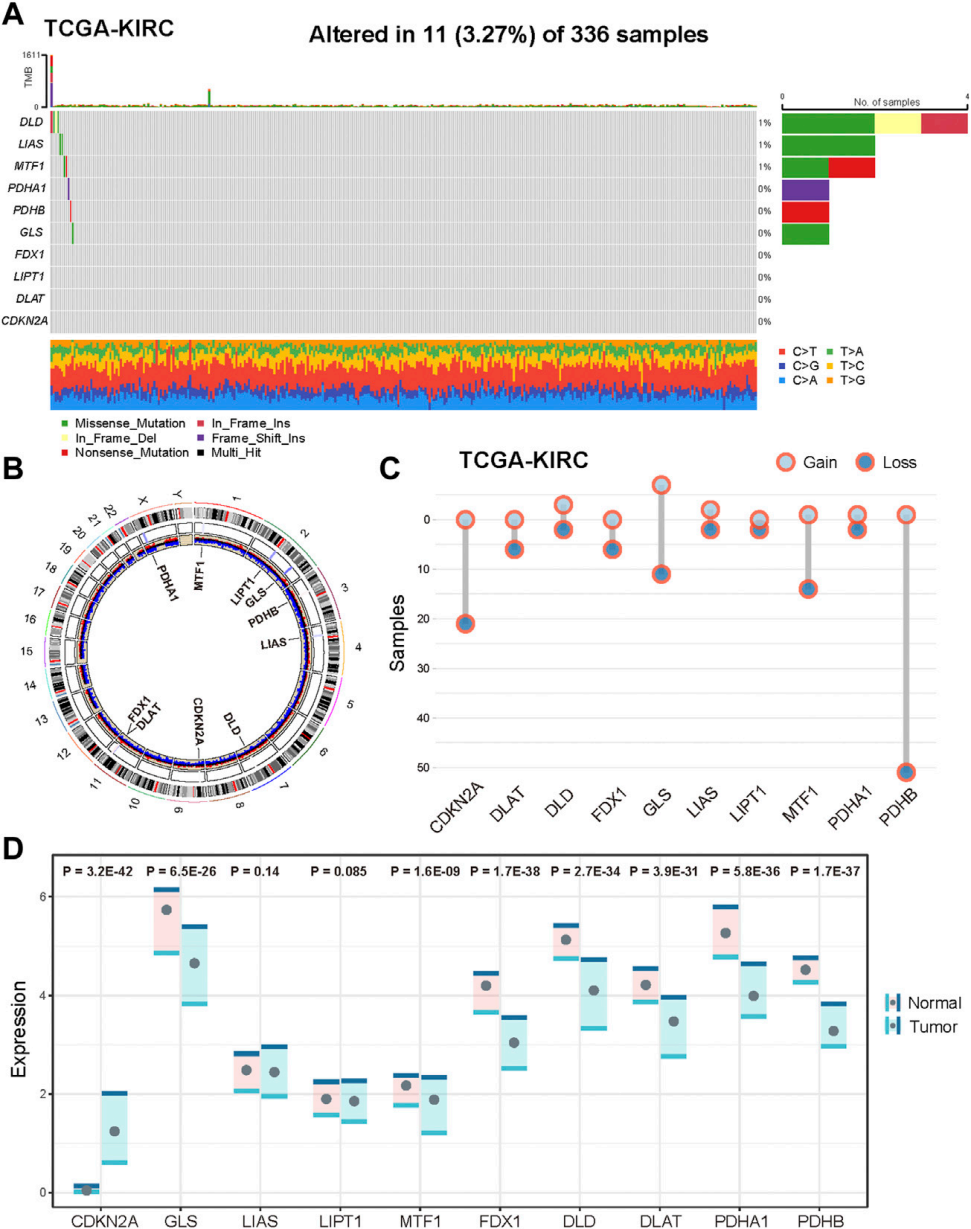
Genetic and transcriptional alterations of CID genes in KIRC. **(A)** Mutation frequencies of CID genes in KIRC patients of TCGA cohort. **(B)** Locations of CNV alterations in CID genes on 23 chromosomes and distribution of expression. **(C)** Frequencies of CNV amplification and deletion of CID genes in TCGA KIRC cohorts. **(D)** Differential expression of CID genes between tumor and normal samples.

The analytical process in this study is illustrated in Supplementary Figure S1. We first explored the landscape of variation in CID genes in genome and transcriptome from TCGA KIRC samples. The incidence of somatic mutations of CID genes is shown in Figure 1A. Among them, DLD had the highest mutation frequency (27%), followed by LIAS, MTF1, GLS, PDHA1, and PDHB. The locations of CNV alterations in CID genes on their respective chromosomes and the expression of CID genes are shown in Figure 1B. PDHB showed the highest frequency of CNV deletion, followed by CDKN2A, MTF1, and GLS. GLS showed the highest frequency of CNV amplification, followed by DLD and LIAS. In addition, we explored the expression levels of CID genes between tumor and normal tissues (Figure 1C). In total, 8 (80%) CID genes showed differential expression, CDKN2A showed significant upregulation, and seven CID genes showed significant downregulation in the tumor samples (Figure 1D, *p* < 0.05, one-sided Wilcoxon rank-sum test).

### Tumor classification based on CID genes

**FIGURE 2:**
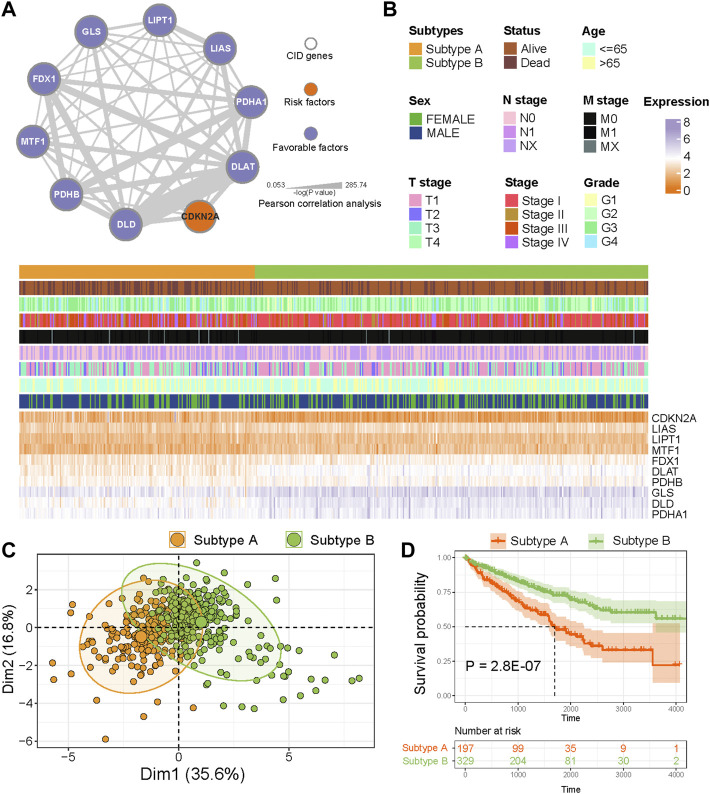
Identification of CID subtypes. **(A)** Co-expression among CID genes in TCGA KIRC cohorts. The line thickness indicate the strength of the correlation. The color of nodes mean prognostic factors of CID genes. **(B)** Two heterogeneous subtypes (subtype A and subtype B) were identified according to unsupervised K-means clustering. **(C)** PCA analysis showing a remarkable difference in expression of CID genes between subtypes. **(D)** Overall survival analysis between subtype A and subtype B.

We explored the prognostic value of CID genes with a univariable Cox proportional hazards regression model. All CID genes were predicted as favorable factors except CDKN2A (Figure 2A). To explore expression correlation among CID genes, we constructed a co-expression network; the thickness of edges means a significance level (Figure 2A). The network indicated a close connection among CID genes.

To analyze the heterogeneity of KIRC, K-means clustering algorithm was used to identify two CID subtypes based on the expression of CID genes (Figure 2B). The PCA revealed that TCGA KIRC samples had distinctive expression patterns of CID genes between two subtypes (Figure 2C). Next, we explored the difference in prognosis between two subtypes; individuals in Subtype A had significantly worse OS when compared with those in Subtype B (Figure 2D, *p* = 2.8 E^−07^, log-rank test).

### Characterization of the immune microenvironment between subtypes

**FIGURE 3:**
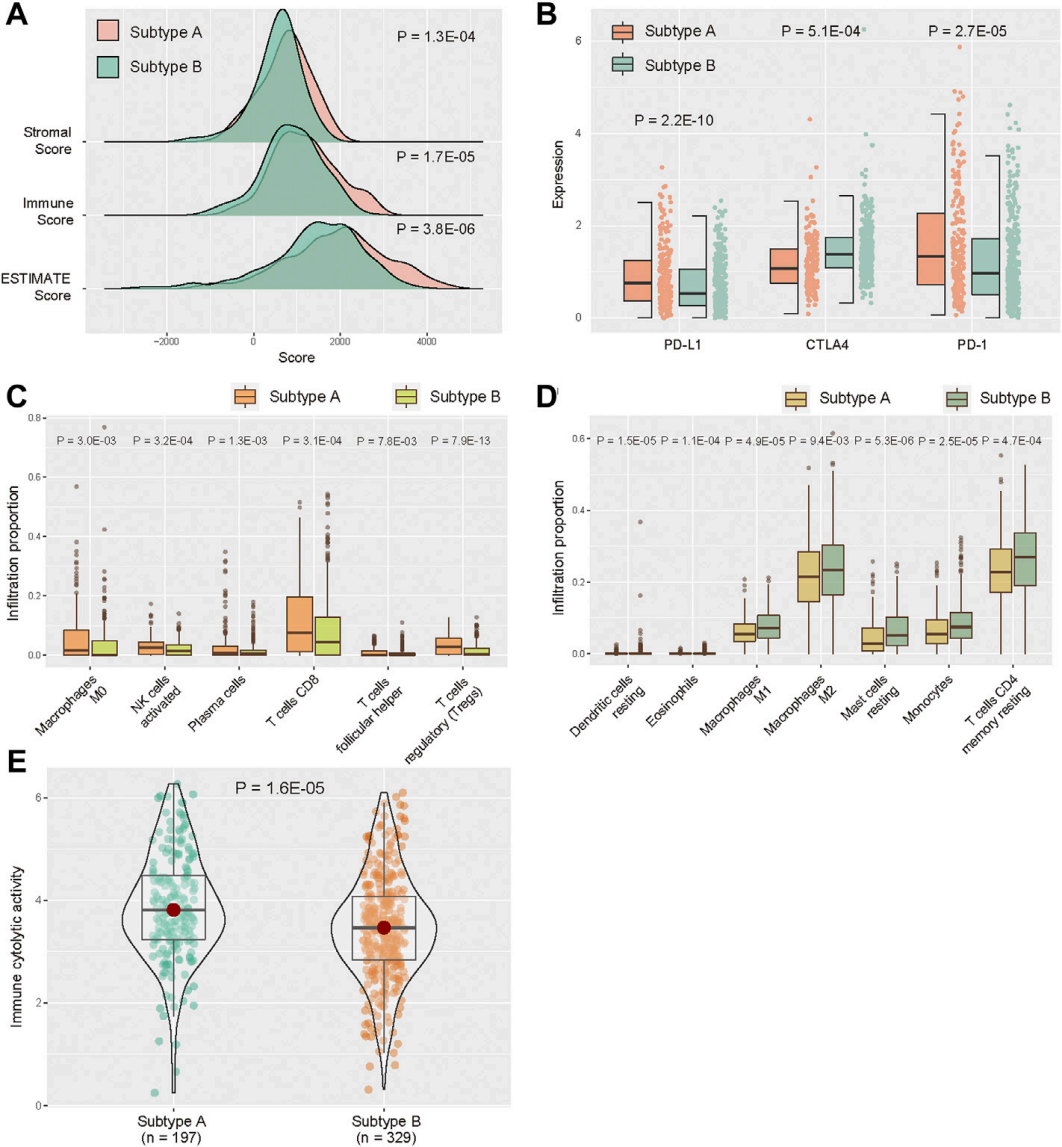
Distribution of TME between subtype A and subtype B. **(A)** Distribution of ESTIMATE score in two subtypes. **(B)** Expression levels of five immune checkpoints between two subtypes. **(C,D)** Abundance of infiltrating immune cell types between two subtypes. **(E)** Distribution of immune CYT score between two subtypes.

The tumor purity distinctions between subtypes, the stromal score, immune score, and ESTIMATE score in Subtype A were significantly higher than those in Subtype B (Figure 3A, *p* < 0.05, one-sided Wilcoxon rank-sum test). Then, we analyzed the differential expression of five immune checkpoints. PD-L1 expression was significantly higher in subtype A compared with that of Subtype B, and the expression of CTLA4 and PD-1 were significantly lower in Subtype A than those in Subtype B (Figure 3B, *p* < 0.05). We also evaluated the distinction of immune cells between two subtypes. According to CIBERSORT algorithm, infiltration of “Macrophages M0,” “NK cells activated,” “Plasma cells,” “T cells CD,” “T cells follicular helper,” and “T cells regulatory (Tregs)” were higher in the Subtype A than those in Subtype B (Figure 3C, *p* < 0.05). Meanwhile, “Dendritic cells resting,” “Eosinophils,” “Macrophages M1,” “Macrophages M2,” “Mast cells resting,” “Monocytes,” and “T cells CD4 memory resting” had significantly lower infiltration in Subtype A compared with Subtype B (Figure 3D, *p* < 0.05). In addition, we evaluated CYT for KIRC samples, and CYT score in Subtype A was higher than that in Subtype B (Figure 3E, *p* < 0.05).

### Identification of DEGs and construction of the prognostic model

**FIGURE 4:**
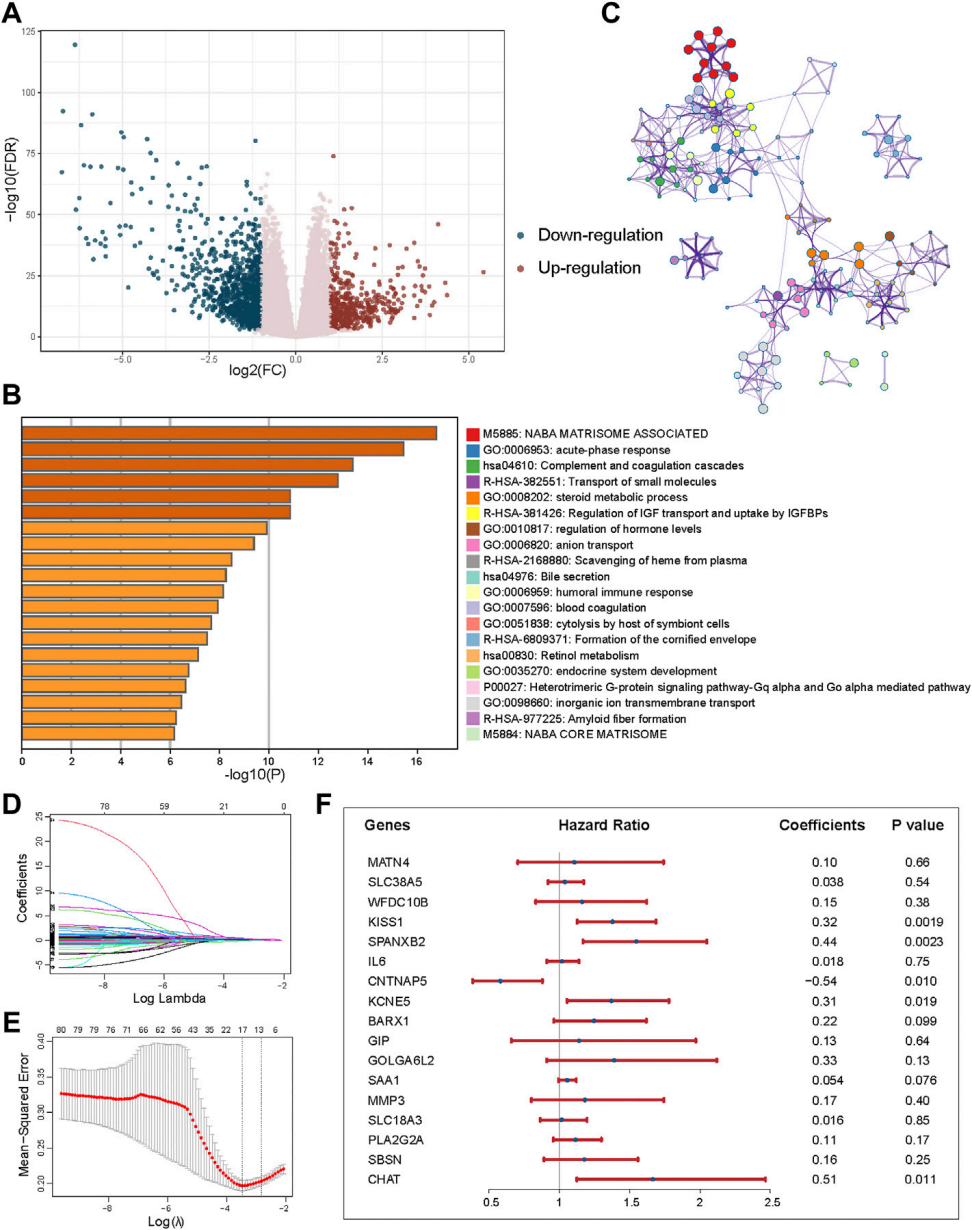
Identification of DEGs between subtypes and construction of the prognostic model. **(A)** Volcano plot showing the differentially upregulated (red points) and downregulated genes (blue points). **(B)** Pathway and process enrichment analysis has been carried out for 344 DEGs that identified between subtypes. The graphical representation showed top 20 enrichments with *p* < 0.01. **(C)** Enrichment terms with a similarity >0.3 are connected by edges. **(D)** LASSO coefficient profiles of 80 prognostic DEGs. **(E)** Cross-validation for tuning parameter selection in the LASSO model. **(F)** Forest plot of the multivariate regression of 17-genes in prognostic model.

To explore the potential biological behavior of CID subtypes, we identified 1448 DEGs between Subtypes A and B (Figure 4A, |log2FC| > 1, FDR <0.001). Pathway and process enrichment analysis for 344 DEGs with |log2FC| > 2 and FDR <0.001 was performed using Metascape tool. DEGs were significantly enriched in “NABA MATRISOME ASSOCIATED,” “acute-phase response,” “Complement and coagulation cascades,” “Transport of small molecules,” and “steroid metabolic process” (Figures 4B,C).

A univariable Cox proportional hazards regression model was performed to identify 80 prognostic DEGs with *p* < 0.001. LASSO method was used for variable selection in a Cox regression model to determine significant prognostic DEGs. One SE above the minimum criteria was chosen, resulting in a model with 17 prognostic genes (Figures 4D,E). Then, based on the expression of the 17 genes, we established a multivariate Cox proportional hazard model (Figure 4F, Supplementary Table S4).

### Validation of the prognostic model

**FIGURE 5:**
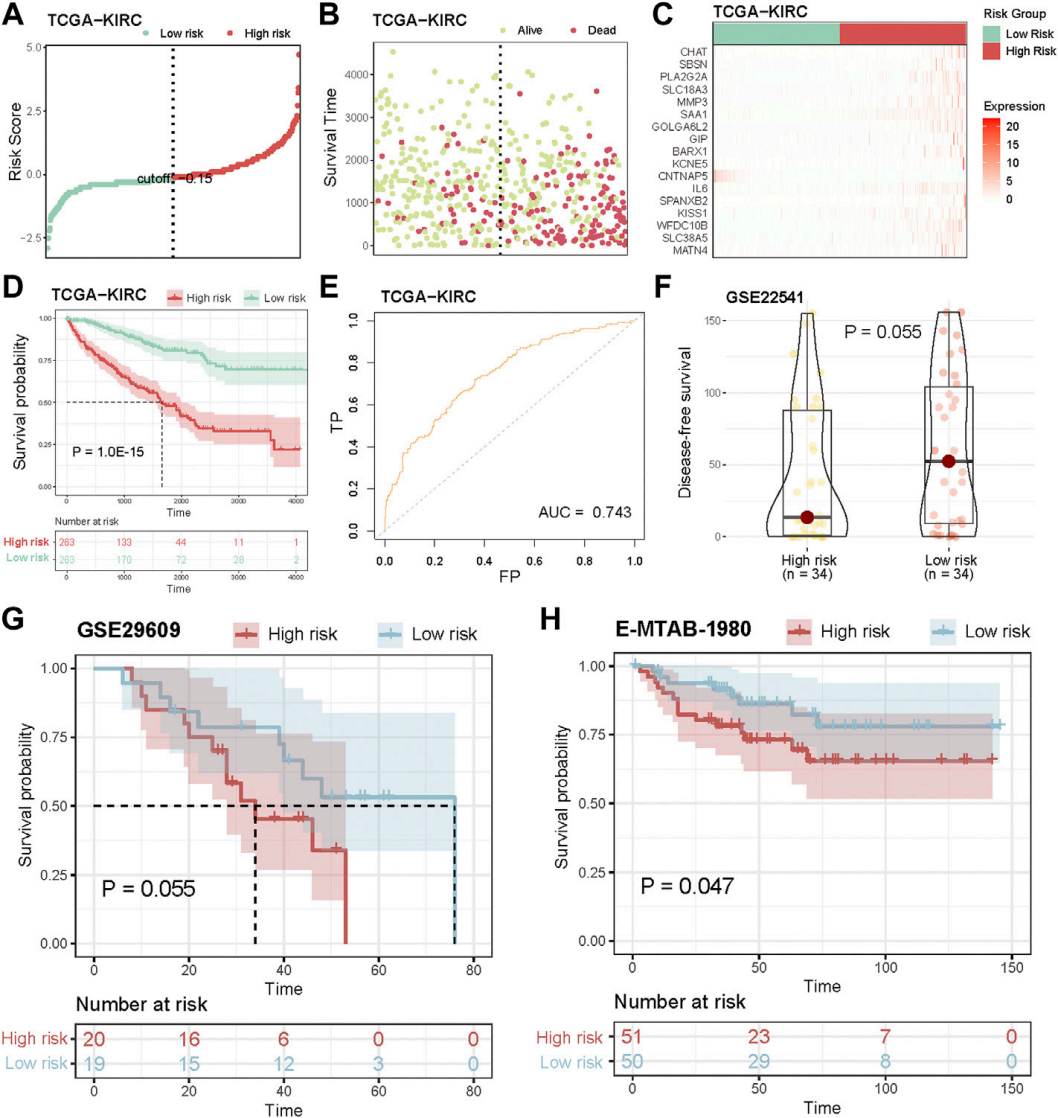
Validation of the prognostic model. **(A)** Ranked dot shows the distribution of risk score. **(B)** Scatter plots shows the distribution of patient survival status. **(C)** Heatmap shows the expression of 17-genes of prognostic model. **(D)** Assessment of the difference in OS between high risk and low risk samples in TCGA cohort by log-rank test. **(E)** ROC curves to predict the sensitivity and specificity of 3-years survival according to the risk score. **(F)** Distribution of DFS between high risk and low risk groups in GSE22541 cohort. **(G,H)** Kaplan-Meier curves show the independent relevance between overall survival time and risk score in GSE29609 and E-MTAB-1980 cohorts.

According to the formula, the risk score of each patient with KIRC was calculated. Patients were classified into the high- and low-risk score groups using the median as the cutoff value (Figure 5A). The distribution plot of the risk scores revealed that survival time decreased while death rates increased with an increase in risk scores in TCGA cohort (Figure 5B). Figure 5C displays the expression of 17 genes in the prognostic model between high and low-risk groups in TCGA (Figure 5C). Furthermore, patients in the high-risk group had a significantly poorer OS (Figure 5D, *p* = 1.0 E^−15^, log-rank test). According to the area under the curve (AUC) of the receiver operating characteristic (ROC) curve, the risk score was able to accurately predict mortality (Figure 5E, AUC = 0.743). Mutations in the tumor suppressor TP53 are associated with various human cancers; consequently, we validated the prognosis power of risk score among TP53 mutation status in TCGA cohort. Patients in the high-risk group had a worse prognosis than patients with TP53 mutation and wild type (Supplementary Figure S2, *p* = 0.092, *p* = 2.5E-14).

Next, we validated the prognosis power of the risk score in independent datasets. In GSE22541 cohort, the disease-free survival (DFS) time of high-risk patients was lower than that of low-risk patients (Figure 5F, *p* = 0.055, log-rank test). Additionally, survival analysis was carried out in two KIRC cohorts (GSE29606 and E-MTAB-1980), and high-risk scores indicated poor prognosis (Figures 5G,H, *p* = 0.055, *p* = 0.047, log-rank test), while the number of surviving patients in the low-risk group were more than those in the high-risk group (Supplementary Figure S3).

**FIGURE 6:**
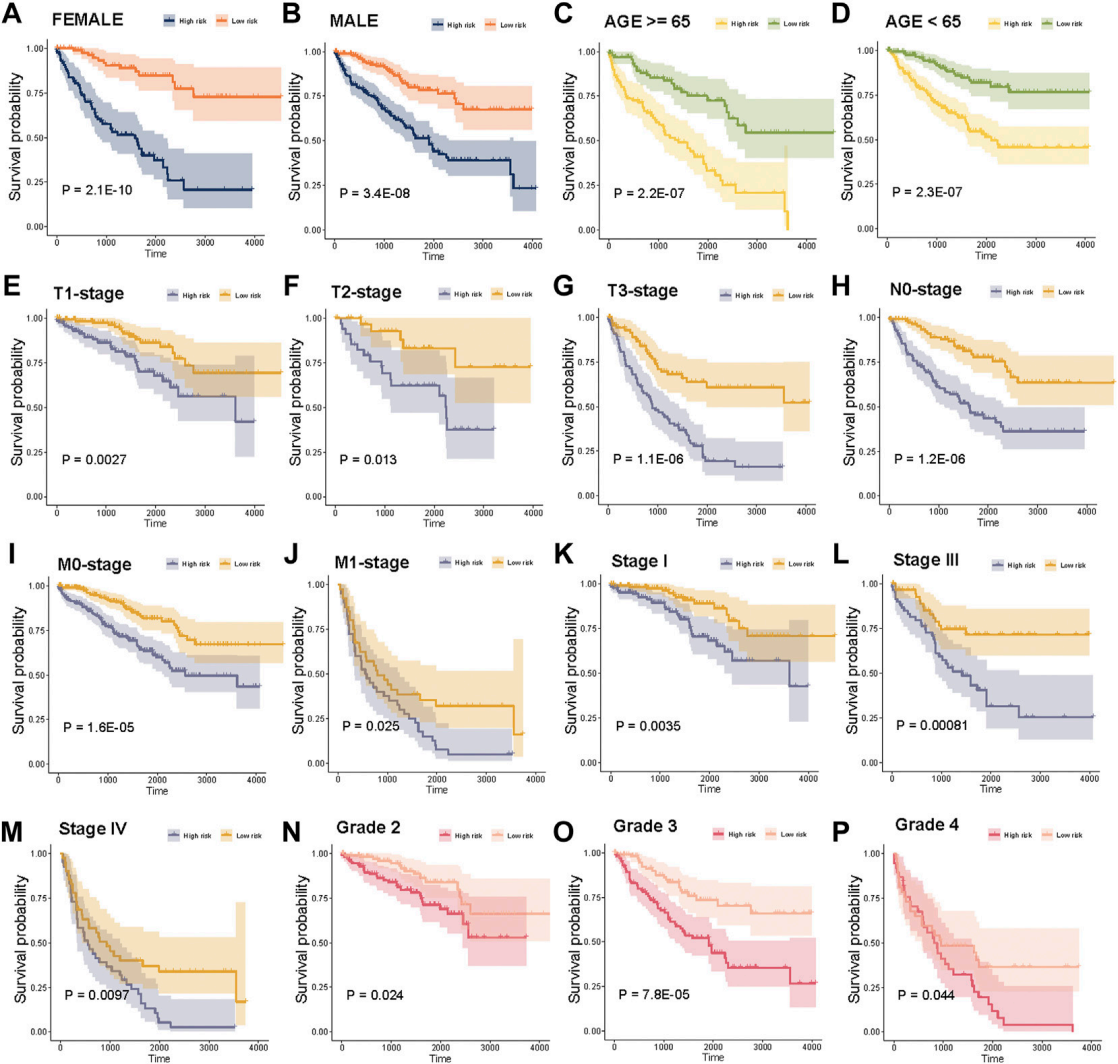
Stratification analysis of the risk score in clinical subgroups. **(A)–(P)** Survival analysis for high risk score and low risk score patients in sex, age, T-stage, N-stage, M-stage, tumor stage, and tumor grade subgroups.

In addition, we explored the power of the risk score to predict the outcome of patients within clinical subgroups. The survival analysis revealed that high-risk score patients had a significantly poorer OS compared with that of the low-risk score patients in females, males, age (≥65), age (<65), T1-stage, T2-stage, T3-stage, N0-stage, M0-stage, M1-stage, Stage I, Stage III, Stage IV, Grade 2, Grade 3 or Grade 4 subgroups (Figure 6, *p* < 0.05, log-rank test).

### Correlation of risk scores and immunotherapy

**FIGURE 7:**
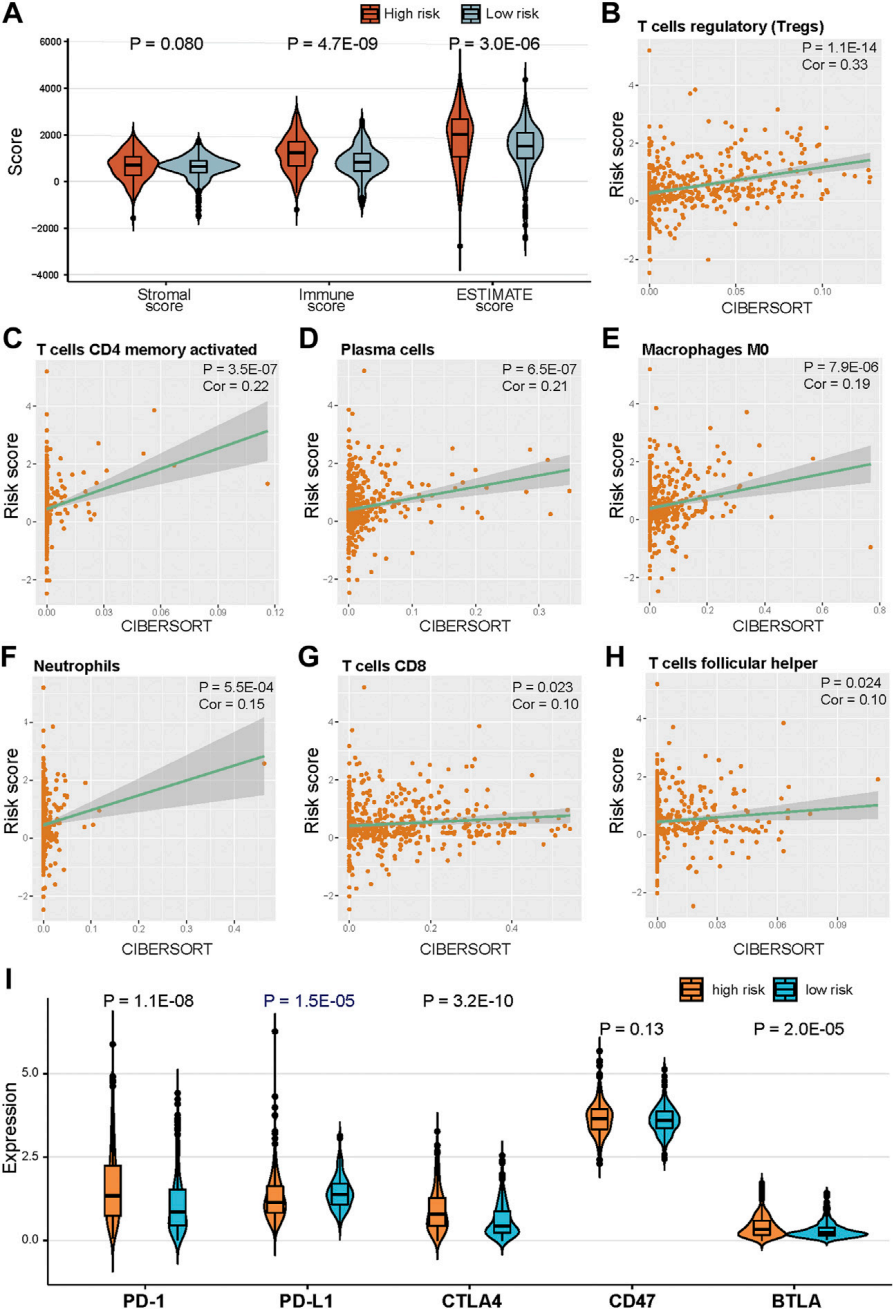
Correlation of risk score and immune cells infiltration. **(A)** Distribution of ESTIMATE score in high risk and low risk groups. **(B)–(H)** Positive correlation between risk score and immune cells. **(H)** Distribution of ESTIMATE score in high risk and low risk groups. **(I)** Expression of five immune checkpoints in high risk and low risk groups.

We further investigated the association between risk scores and immune infiltration. The stromal, immune, and ESTIMATE scores of high-risk score samples were higher than those in low-risk score samples (Figure 7A). The Pearson correlation analysis was used to assess the correlation between risk scores and the abundance of immune cells. Infiltration of “T cells regulatory (Tregs),” “T cells CD4 memory activated,” “Plasma cells,” “Macrophages M0,” “Neutrophils,” “T cells CD8,” and “T cells follicular helper” were significantly positively correlated with-risk scores (Figures 7B–H, *p* < 0.05, Pearson correlation analysis). We also assessed the relationship between the expression of five immune checkpoints and the risk score. PD-1, CTLA4, and BTLA expressions were significantly higher in high-risk samples compared with low-risk samples in TCGA KIRC cohort, and PD-L1 expression was significantly lower in high-risk samples (Figure 7I, *p* < 0.05).

**FIGURE 8:**
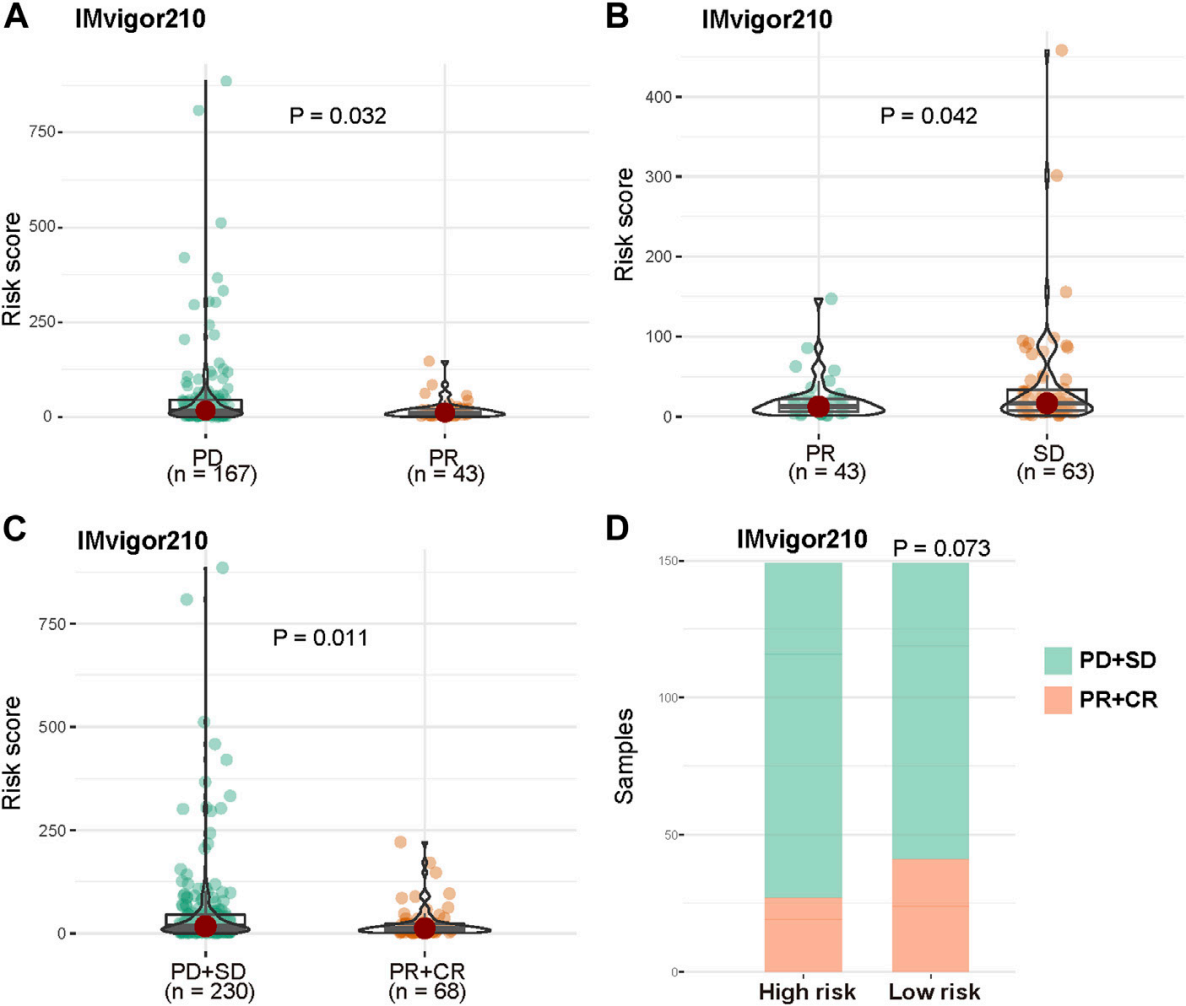
Prognosis power of risk score in patients with immunotherapy. **(A)** Distribution of risk score between patients with PD and PR. **(B)** Distribution of risk score between patients with PR and SD. **(C)** Distribution of risk score between non-responsive (PD and SD) patients and responsive (PR and CR) patients in IMvigor210 cohort. **(D)** Distribution of non-responsive and responsive patients between high risk and low risk groups.

To further explore if the risk score can predict patients’ response to immunotherapy, we evaluated the risk score differences among patients in immunotherapy response subgroups. The risk score of patients with progressive disease (PD) or stable disease (SD) was significantly higher compared with patients with partial response (PR) (Figures 8A,B, *p* = 0.032, *p* = 0.042). Moreover, the risk score of non-responsive (PD and SD) patients were significantly higher than that of responsive (PR and complete response [CR]) patients (Figure 8C, *p* < 0.011). The numbers of patients in the high-risk group with PD and SD were more than those in the low-risk group; PR and CR patients were more abundant in the low-risk group (Figure 8D, *p* = 0.073, hypergeometric test).

## Discussion

In this study, we analyzed the role of CID in ccRCC progression, microenvironment alteration, and clinical prognosis. When analyzing the somatic mutation status of CID-related genes in ccRCC, most of them have a high mutation frequency. Meanwhile, the difference in CID-related gene expression is significant in cancer and normal tissues. These results further implied the potential of CID to be developed as a cancer therapy target and prognosis indicator (Ge et al., 2022; Oliveri, 2022).

Accordingly, we then identified ccRCC subtypes based on CID-related genes with a K-means clustering algorithm. Copper and CID plays the essential role in the mudutlation of cancer immune microenvironment. For example, recent research demonstrated that major copper influx transporter copper transporter 1 is correlated with PD-L1 expression across many cancer types (Voli et al., 2020). Meanwhile, copper chelators play the role in the inhibition of STAT3 and EGFR’s phosphorylation and promoted the degradation of PD-L1 (Voli et al., 2020). Further, copper in also correlated with the cancer’s immunogenic cell death in breast cancer (Kaur et al., 2020). Significant immune heterogeneity across the two ccRCC subtypes was observed. In Subtype B, ccRCC has the highest ESTIMATE score, infiltration level of CD8^+^ T cell and NK cell, and immune cytolytic activity. This result indicated that Subtype B may have higher immune activity. In general, the high immune activity of cancer implies a better prognosis (Chen and Mellman, 2017). However, the immune activity of ccRCC subtype B has a worse prognosis than subtype A. Xu et al. (2019) also indicated that high immune activity relates to poor prognosis. Nakano et al. (2001) also found that a high infiltration level of CD8^+^ T cells correlates with a poor RCC prognosis. Clonal variation of immune cells of the microenvironment may contribute to this unique characteristic of ccRCC (Borcherding et al., 2021).

Next, we acquired DEGs between Subtypes A and B and conducted the enrichment analysis to reveal the role of CID in ccRCC. According to the results, DEGs concentrated on the immune and metabolic-related processes. Cell toxicity mediated by copper was correlated with glucose metabolism activity (Li et al., 2022). Glucose metabolism alterations of microenvironment components, including cancer cells and immune cells, leading to the formation of different tumor subtypes (Li and Zhang, 2016; Terrén et al., 2019; Zhang et al., 2021). Our research demonstrated that CID may contribute to the immune microenvironment heterogeneity in ccRCC. Consequently, further analysis concentrating on the detailed interaction of CID-mediated metabolism alteration and TME may illustrate the regulatory function of CID in cancer.

Based on the obtained DEGs, we constructed a model to predict the prognosis of patients with ccRCC. Our result demonstrated that the risk score can greatly predict patient prognosis in training and validation datasets. Furthermore, the risk score is effective in different stages (i.e., T, N, and M) and grades. Accordingly, our risk score offers great clinical applicability.

Immunotherapy is widely applied in treating different types of solid cancer (Helmy et al., 2013; Robert et al., 2015; Larkin et al., 2019). Therefore, we further explored the potential of our risk score for predicting the immune features and therapy responsiveness of cancer. High-risk scores predict the lower expression level of PD-L1. Meanwhile, in the anti-PD-L1 cohort, high-risk scores are correlated with a low therapy responsiveness rate. Consequently, anti-PD-L1 therapy may be a suitable choice for low-risk score patients.

It is worth noting the limitations of the research. First, large-scale multi-omics immunotherapy data should be employed to more comprehensively evaluate the potential of the risk score for predicting immunotherapy responsiveness. Due to the lack of high-quality validated data, the enrolled immunotherapy samples are limited. Second, combining the transcriptome analysis of the clinical samples and follow-up data will further test the robustness of the risk score. Third, *in vivo* and *in vitro cell*-line and animal models may help explore the potential underlying mechanism of CID in cancer. These shortcomings will be overcome with the rapid progression of big data and our further in-depth research.

In summary, our research revealed the role of CID in ccRCC, identified ccRCC subtypes based on CID features and constructed a robust gene prognostic model to predict patient prognosis. Our research laid a foundation for CID-related analysis and presented a prognostic model which can be potentially applied in the clinical treatment of ccRCC.

## Data Availability

Publicly available datasets were analyzed in this study. The names of the repository/repositories and accession number(s) can be found in the article/Supplementary Material.

## Abbreviations

ccRCC, clear cell renal cell carcinoma; CID, copper ionophore–induced death; CNV, copy number variation; CR, complete response; CYT, cytolytic activity; DEGs, differentially expressed genes; DFS, disease-free survival; TCGA, The Cancer Genome Atlas; GEO, Gene Expression Omnibus; LASSO, Least Absolute Shrinkage and Selection Operator; RCC, renal cell carcinoma; TCA, tricarboxylic acid; KIRC, kidney clear cell carcinoma; mUCC, metastatic urothelial cancer; OS, overall survival; PCA, principle component analysis; SE, standard error; TME, tumor immune microenvironment; Tregs, T cells regulatory; PD, progressive disease; SD, stable disease; PR, partial response; UCSC, the University of California Santa Cruz.
