# Supplementary material for: Role of copper ionophore–induced death in immune microenvironment and clinical prognosis of ccRCC: An integrated analysis
Source: Front Genet. 2022 Oct 3;13:994999. doi: 10.3389/fgene.2022.994999 (PMC9574041; doi:10.3389/fgene.2022.994999)
Supplement: Supplementary file 3 [file Table1.DOCX]

**Table S1. Baseline characteristics of patients in TCGA KIRC cohort**

| **Characteristics** | **Whole cohort** | **High PSR_score** | **Low PSR_score** | ***p*** |
| --- | --- | --- | --- | --- |
| TCGA cohort | (n=526) | (n=263) | (n=263) |  |
| Gender |  |  |  | 0.044 |
| Male | 342(65.02%) | 182(69.2%) | 160(60.84%) |  |
| Female | 184(34.98%) | 81(30.8%) | 103(39.16%) |  |
| Age |  |  |  | 0.087 |
| <65 years | 329(62.55%) | 155(58.94%) | 174(66.16%) |  |
| >=65 years | 197(37.45%) | 108(41.06%) | 89(33.84%) |  |
| T-stage |  |  |  | 9.1e-11 |
| T1 | 269(51.14%) | 95(36.12%) | 174(66.16%) |  |
| T2 | 68(12.93%) | 36(13.69%) | 32(12.17%) |  |
| T3 | 178(33.84%) | 121(46.01%) | 57(21.67%) |  |
| T4 | 11(2.09%) | 11(4.18%) | 0(0%) |  |
| N-stage |  |  |  | 0.0023 |
| N0 | 239(45.44%) | 115(43.73%) | 124(47.15%) |  |
| N1 | 16(3.04%) | 14(5.32%) | 2(0.76%) |  |
| M-stage |  |  |  | 2.0e-10 |
| M0 | 436(82.89%) | 191(72.62%) | 245(93.16%) |  |
| M1 | 80(15.21%) | 66(25.1%) | 14(5.32%) |  |
| Stage |  |  |  | 3.1e-06 |
| I | 263(50%) | 91(34.6%) | 172(65.4%) |  |
| II | 56(10.65%) | 28(10.65%) | 28(10.65%) |  |
| III | 122(23.19%) | 75(28.52%) | 47(17.87%) |  |
| IV | 82(15.59%) | 68(25.86%) | 14(5.32%) |  |
| Grade |  |  |  | 1.2e-05 |
| G1 | 14(2.66%) | 1(0.38%) | 13(4.94%) |  |
| G2 | 224(42.59%) | 81(30.8%) | 143(54.37%) |  |
| G3 | 205(38.97%) | 112(42.59%) | 93(35.36%) |  |
| G4 | 75(14.26%) | 67(25.48%) | 8(3.04%) |  |
