# Supplementary material for: Role of copper ionophore–induced death in immune microenvironment and clinical prognosis of ccRCC: An integrated analysis
Source: Front Genet. 2022 Oct 3;13:994999. doi: 10.3389/fgene.2022.994999 (PMC9574041; doi:10.3389/fgene.2022.994999)
Supplement: Supplementary file 6 [file DataSheet1.DOCX]

**Supplementary Information**

**Supplementary Figures**

**
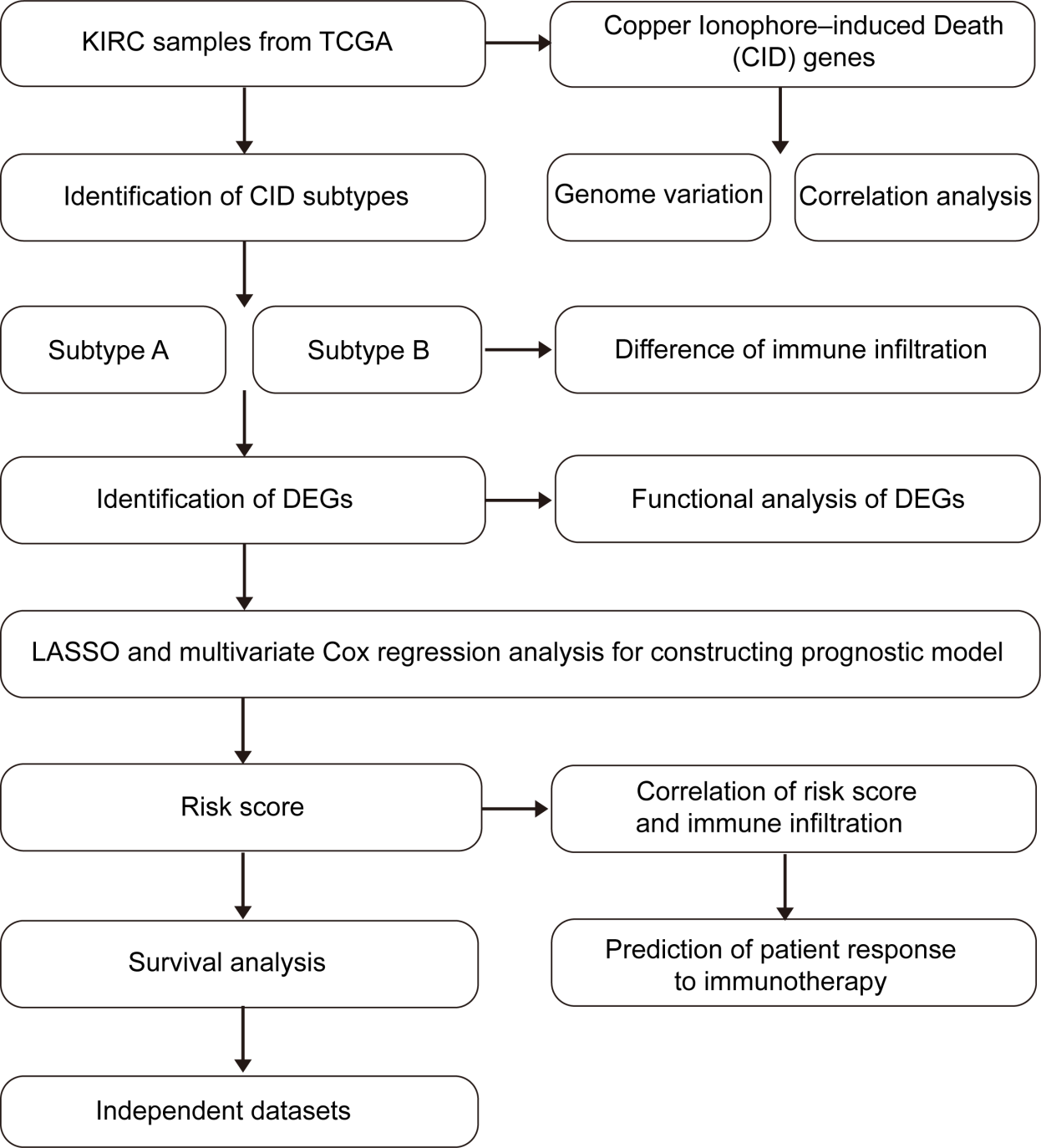
**

**Figure S1. The analytical process in this study.**


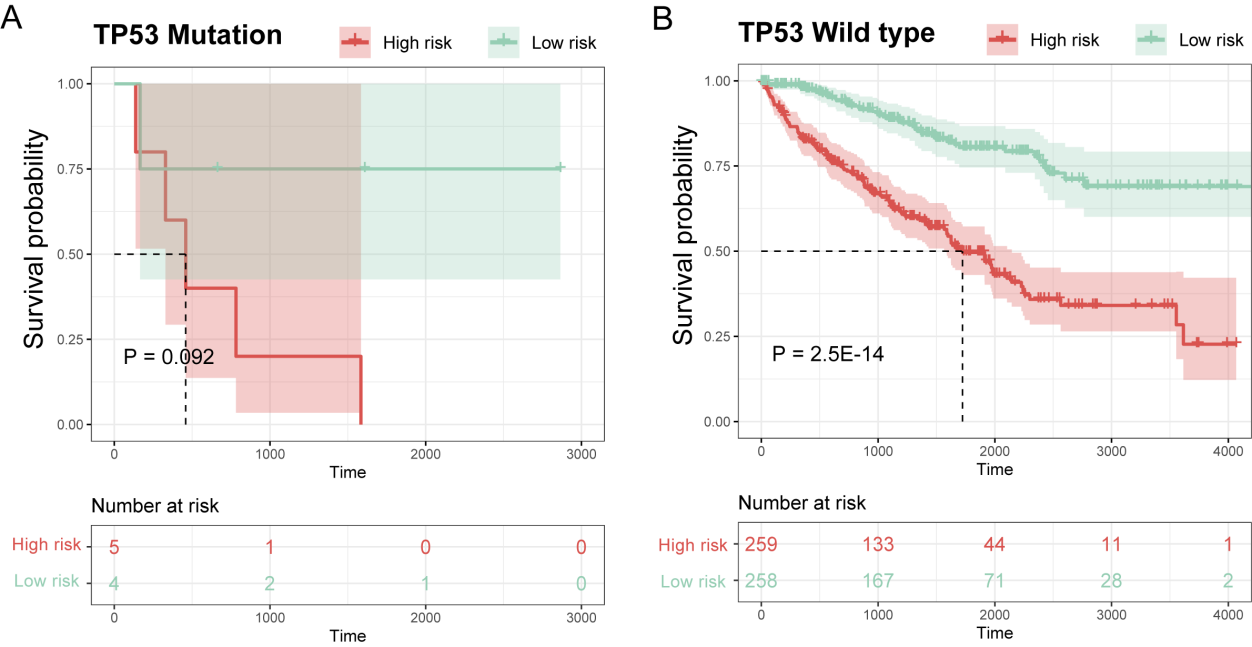


**Figure S2. Prognosis power of risk score among TP53 mutation status.**

**(A)** Survival analysis of high risk and low risk patients with TP53 mutation. **(B)** Survival analysis of high risk and low risk patients with TP53 wild type.


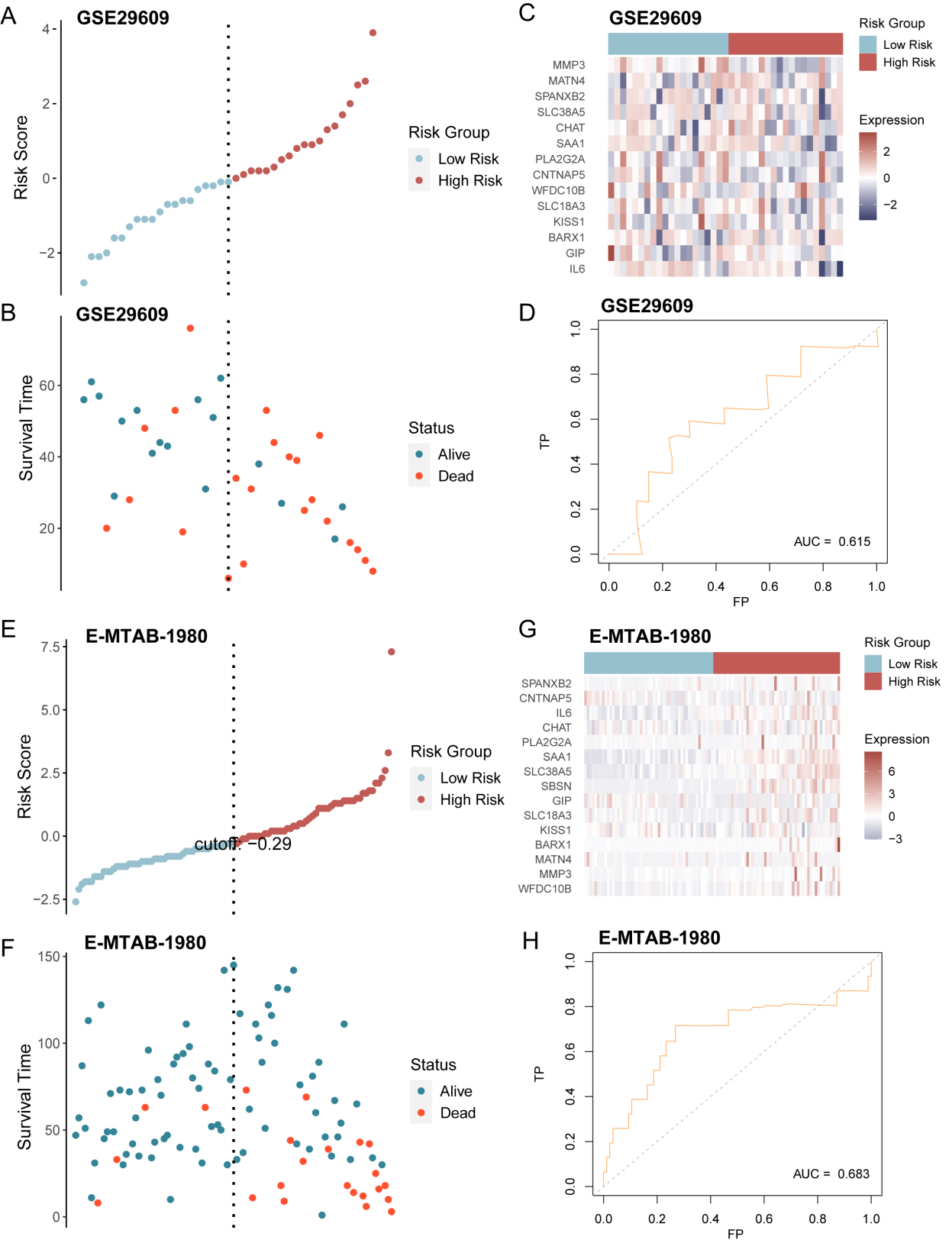


**Figure S3. Validation of the prognosis power of risk score in independent datasets.**

**(A)-(B)** Distribution of risk score and patient survival status in GSE29609 cohort. **(C)** Heatmap shows the expression of 14 genes in prognostic model between risk groups in GSE29609 cohort. **(D)** ROC curves to predict the sensitivity and specificity of 3-year survival according to the risk score in GSE29609 cohort. **(E)-(F)** Distribution of risk score and patient survival status in E-MTAB-1980 cohort. **(G)** Heatmap shows the expression of 15 genes in prognostic model between risk groups in E-MTAB-1980 cohort. **(H)** ROC curves to predict the sensitivity and specificity of 3-year survival according to the risk score in E-MTAB-1980 cohort.
